# Pirfenidone attenuates bleomycin-induced pulmonary fibrosis in mice by regulating Nrf2/Bach1 equilibrium

**DOI:** 10.1186/s12890-017-0405-7

**Published:** 2017-04-18

**Authors:** Yuan Liu, Fuai Lu, Lirong Kang, Zhihua Wang, Yongfu Wang

**Affiliations:** Department of Rheumatology, First Affiliated Hospital of Baotou Medical College, Inner Mongolia University of Science & Technology, Baotou, Inner Mongolia 014010 China

**Keywords:** Idiopathic pulmonary fibrosis, Pirfenidone, Oxidative stress, Nrf2/Bach1

## Abstract

**Background:**

Oxidative stress is one of the important factors involved in the pathogenesis of idiopathic pulmonary fibrosis (IPF). The equilibrium of Nuclear factor-erythroid-related factor 2 (Nrf2)/[BTB (broad-complex, tramtrack and bric-a-brac) and CNC (cap‘n’collar protein) homology 1, Bach1] determines the expression level of antioxidant factors, further regulating the function of oxidation/antioxidation capacity. Pirfenidone (PFD) is one of two currently for IPF therapy approved drugs. PFD regulates intracellular antioxidants, inhibits secretion of inflammatory cytokines and collagen synthesis. However the mechanisms of its antioxidant effects remain elusive.

**Methods:**

Effects of PFD treatment were studied in mouse lung fibroblasts (MLF) following induction by transforming-growth factor beta 1 (TGF-β1) and in mice following bleomycin-induced lung fibrosis. The mRNA and protein levels of oxidative stress-related factors Nrf2/Bach1 and their downstream antioxidant factors heme oxygenase-1 (Ho-1) and glutathione peroxidase 1 (Gpx1) were determined by RT-PCR and Western blot. Fibrosis-related cytokines interleukin-6 (IL-6) and myofibroblast markers type 1 collagen α1 (COL1A1) levels in supernate of MLF, serum, and bronchoalveolar lavage fluid (BALF) as well as malondialdehyde (MDA) in serum and BALF were detected by ELISA, reactive oxygen species (ROS) generation was measured by 2′,7′- dichlorofluorescin diacetate (DCFH-DA) assay and lung pathological/morphological alterations in mice were observed by HE and Masson to assess the antioxidant mechanism and therapeutic effects on pulmonary fibrosis induced by bleomycin.

**Results:**

PFD inhibited Bach1 mRNA and protein expressions in mouse lung fibroblasts induced by TGF-β1 and lung tissues with pulmonary fibrosis induced by bleomycin. Furthermore, it improved Nrf2, Ho-1 and Gpx1 mRNA and protein expressions. After PFD treatment, COL1A1and IL-6 levels in supernate of MLF, serum, and BALF as well as ROS in lung tissues and MDA in serum and BALF from a mouse with pulmonary fibrosis were significantly decreased, and the infiltration of lung inflammatory cells and fibrosis degree were alleviated.

**Conclusions:**

Theraputic effects of PFD for IPF were involved in Nrf2/Bach1 equilibrium which regulated the capacity of oxidative stress. The study provided new insights into the antioxidant mechanism of PFD.

**Electronic supplementary material:**

The online version of this article (doi:10.1186/s12890-017-0405-7) contains supplementary material, which is available to authorized users.

## Background

Idiopathic pulmonary fibrosis (IPF) is a disease characterized by diffused alveolar inflammation and extensive interstitial fibrosis, which is related with various cells, inflammatory factors, fibrosis related cytokines, and transforming growth factor-β1 (TGF-β1) signal transduction [1, 2]. It leads to high mortality, and the average survival time after diagnosis is about 2–5 years [3]. TGF-β1 is a key factor for the initiation of pulmonary fibrosis that promotes proliferation and transformation of lung fibroblasts and deposition of extracellular matrix [4]. Nonetheless, the pathogenesis of IPF is not entirely understood, and hence, an efficient treatment is rather limited [5, 6]. Therefore, it is vital to investigate the pathogenesis and therapeutic mechanisms of IPF.

Pirfenidone (PFD) is approved drug for IPF treatment with anti-fibrotic, antioxidant, and anti-inflammatory effects [7]. It inhibits the differentiation and proliferation of fibroblasts as well as the synthesis of collagen and generation of inflammatory cytokines [8, 9]. The clinical study indicated that PFD effectively improved the pulmonary function and quality of life for patients with IPF [10]. Moreover, it was found that PFD could also clear oxygen free radicals and exert anti-fibrosis effect [11]. In addition, it stimulated the generation of reduced glutathione (a major antioxidant substance), increase oxidative stress capacity, and exert antioxidant effects [12]. Despite that the exploration of the antioxidant mechanism of PFD is not intensive.

Oxidative stress and oxidation/antioxidation imbalance play a major role in the pathogenesis and progression of IPF [13, 14]. Nuclear factor-erythroid-related factor 2 (Nrf2) belongs to cap ‘n’ collar (CNC) transcription family that regulates the expression of a wide array of antioxidant genes. BTB (broad-complex, tramtrack and bric-a-brac) and CNC (cap‘n’collar protein) homology 1, Bach1 has been regarded as a competitive inhibitor for Nrf2 by intervening and competitively inhibiting the interaction of Nrf2 and antioxidant response element (ARE). This negatively regulates the downstream antioxidant genes including heme oxygenase-1 (Ho-1) and glutathione peroxidase 1 (Gpx1) [15–17]. It was found that Nrf2 and its downstream antioxidant factors are involved in the pathogenesis of IPF [18]. Nrf2 agonist attenuated pulmonary fibrosis induced by bleomycin (BLM) via the oxide level in lung tissue [19]. Our previous study demonstrated that Bach1 knockout inhibited the progression of BLM-induced pulmonary fibrosis by regulating the expressions of Nrf2 and its downstream anti-oxidant factors including Ho-1 and Gpx1, thus providing novel insights into the role of Bach1/Nrf2 in the regulation of oxidative stress involved in the pathogenesis of IPF [20].

Based on its antioxidative property of Nrf2/Bach1 in oxidative stress regulation, PFD was used to intervene mouse lung fibroblasts (MLF) induced by TGF-β1 and mice with pulmonary fibrosis induced by BLM in order to observe the influences of the drug on expressions of Nrf2/Bach1 and the downstream antioxidant factors, Ho-1 and Gpx1. Furthermore, the effects of PFD on fibrosis-related cytokines interleukin-6 (IL-6), myofibroblast markers type 1 collagen α1 (COL1A1), common indicators of oxidative stress such as reactive oxygen species (ROS) and malondialdehyde (MDA) in mouse and lung pathological alterations were also investigated. The present study explores the underlying antioxidative mechanism and therapeutic efficacy of PFD on IPF.

## Methods

### Experimental materials

C57BL/6 mice were acquired from Vital River Laboratories (Beijing, China) and primary mouse lung fibroblasts (MLF), MIC-CELL-0040 from PriCells (Hubei, China). RNA extraction kit was purchased from Tiangen Biotech (Beijing, China), reverse transcription kit and fluorescent quantitation PCR kit from Takara (Tokyo, Japan). Nrf2, Bach1, HO-1, GPx1 rabbit anti-mouse primary antibodies were purchased from Abcam (Cambridge, MA, USA). Protein Extraction Kit was obtained from Sigma-Aldrich (St. Louis, MO, USA) and ROS kit were purchased from Jiancheng Bioengineering Institute (Nanjing, China). Enzyme linked immunosorbent assay (ELISA) Kit was obtained from R&D (R&D Systems, Minneapolis, MN, USA) and Cloud-Clone Corp (USCN Life Science Inc, Wuhan, China). Primers were synthesized by Sangon (Shanghai, China). Hematoxylin staining kit and Masson trichrome staining kit were purchased from Solarbio (Beijing, China). TGF-β1 recombinant protein was obtained from R&D, and PFD was obtained from Shionogi & Co., Ltd (Osaka, Japan).

### Animals and administration

7–8 week old male C57BL/6 mice (body weight 18–22 g) were maintained under specific pathogen-free conditions and acclimated for 1 week at 22 ± 3 °C and humidity (45–60%) in a 12:12 h light before the experiments. The mice were permitted ad libitum access to food and water. Fifteen mice were randomly divided into 5 equal groups: control group (50 μL 0.9% saline, administered intratracheally once), BLM 14d group (14d after 5.0 mg/kg BLM dissolved in saline to 50 μL, intratracheally injected once, at the time of establishment of lung fibrosis), BLM 42d group (at the final time point of administration), only PFD group (300 mg/kg PFD, orally administrated daily for 4 weeks) and BLM + PFD group (300 mg/kg PFD treatment for 4 weeks after 14d of BLM administration). We assessed the model of pulmonary fibrosis after 14d and 42d of BLM administration. At the designated endpoint, serum and BALF of mice were collected. Then the mice were sacrificed, and lung tissues excised to identify pulmonary inflammation and fibrosis by pathological morphology. The lung lavage was obtained by washing the lung three times with saline (0.9%) through a tracheal cannula. Post-execution, both the lung tissues were harvested, and a part of the pulmonary lobe was preserved in liquid nitrogen after being treated by diethylpyrocarbonate (DEPC); others were fixed in 4% formaldehyde. The animal experiments were approved by the Ethics Committee of Baotou Medical College (201511306).

### Cell culture and intervention

Primary mouse lung fibroblasts were cultured in DMEM medium containing 10% FBS, 100 U/mL penicillin and 100 U/mL streptomycin at 37 °C in a 5% CO_2_-humidified atmosphere. At 70–80% confluency, the cells were seeded at 1 × 10^5^/mL in a 6-well plate. After serum starvation for 24 h, they were treated by 5 ng/mL TGF-β1 for 24 h followed by stimulation with 100, 200 and 500 μg/mL PFD, was added to the supernatant for an additional 48 h. In all in vitro experiments, gene expression (transcript and protein) was assessed at the same time point, 72 h after TGF-β1 treatment. At the final point of TGF-β1 and various concentrations of PFD administration, the cells and supernates were harvested respectively. Three biological replicates were performed, and experiments repeated three time for each.

### RT–PCR

Total RNA from the fibroblasts and mouse lung tissues was extracted by RNA extraction kit, and cDNA was synthesized by reverse transcription according to the manufacturer’s instructions. The relative expression level of mRNA was detected by the PCR kit. The primer sequences designed by Primer Premier 5.0 are shown in the following Additional file 1: Table S1. Reverse transcription was carried out in a total volume of 20 μL at 65 °C for 5 min, 42 °C for 60 min, and 70 °C for 15 min. The PCR conditions (ABI Prism 7500, Applied Biosystems) were as follows: 95 °C, 30 s; 60 °C, 1 min; 72 °C, 30 s for 40 cycles. The mouse GAPDH was used as internal control, and 2^–ΔΔCT^ was used to calculate the relative expression level of the target genes. The experiments were performed at least 3 times (Table 1).

**Table 1:** PCR primers sequence

Nrf2	Forward (5′- 3′)	GTGGTTTAGGGCAGAAGG
	Reverse (5′- 3′)	TCTTTCTTACTCTGCCTCTA
Bach1	Forward (5′- 3′)	ACAGGGCTACTCGCAAA
	Reverse (5′- 3′)	GTCATCTCCCAGGCTAATC
HO-1	Forward (5′- 3′)	GACAGAAGAGGCTAAGACCGC
	Reverse (5′- 3′)	TGACGAAGTGACGCCATCT
GPx1	Forward (5′- 3′)	GCACATCTACCACGCAGTCA
	Reverse (5′- 3′)	AGAGTCTCAAGAACATCGCCT
GAPDH	Forward (5′- 3′)	AAGACCCAGAAATGAAC
	Reverse (5′- 3′)	TCTACACGATAACAACCA

### Western blot

Mouse lung tissues and lung fibroblasts were lysed for protein extraction according to the manufacturer’s instructions. The protein concentration was evaluated by BCA method. The total protein was transferred onto a nitrocellulose membrane after resolving on 10% SDS-PAGE and blocked for 1 h with 5% skim milk, Subsequently, the membrane was probed with primary antibodies, such as rabbit anti-mouse Nrf2 (1:500), Bach1 (1:300), Ho-1 (1:500), Gpx1 (1:1000), and β-actin (1:500), respectively, at 4 °C overnight. Then, secondary antibody labeled with horseradish peroxidase (1:10000) was added for 1 h at room temperature and protein bands were visualized by using double infrared laser scanning imaging system (Licor, USA).

### ELISA

IL-6 and COL1A1 levels in supernate of lung fibroblasts, serum, and BALF as well as MDA expression in serum and BALF were performed by ELISA kit according to the manufacturer’s instructions.

### Chemiluminescence

Single cell suspensions were obtained from lung tissues of mice by enzymatic dissociation and the number of cells counted no less than 1 × 10^6^. ROS were measured using the 2′,7′- dichlorofluorescin diacetate (DCFH-DA) assay. The cells were incubated with 10 μM DCFH-DA for 30–60 min at 37 °C and then were washed with PBS. Fluorescence density was measured by FACScan instrument (Becton Dickinson, San Jose, CA, USA) at the excitation wavelength of 485 nm and the emission wavelength of 530 nm.

### Pathological morphology

Lung tissues were fixed with 4% formalin for 12–16 h, then dehydrated and embedded in paraffin. The sample blocks were sliced into 5 μm and stained by HE to observe the inflammatory infiltration and integrity of the alveolar structure. Masson trichrome was used to assess the amount of fibrosis in the lung. The degree of pulmonary alveolitis was scored according to Szapiel et al. [21], and pulmonary fibrosis by Ashcroft score [22].

### Statistics

Statistical analysis was performed by SPSS 17.0, and the data were expressed as mean ± SD. Comparison among groups was analyzed by one-way analysis of variance (ANOVA), and *P* < 0.05 was statistically significant.

## Results

### Effects of PFD on the expressions of oxidants/antioxidants in MLF stimulated by TGF-β1

**Fig. 1:**
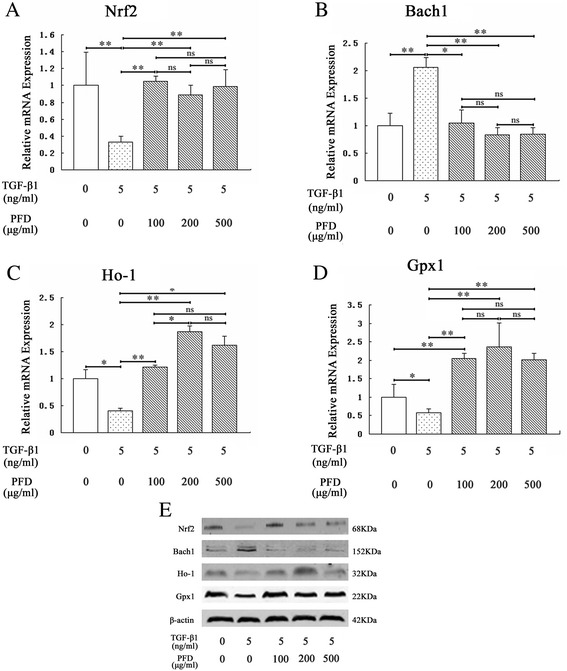
Expression of oxidant/antioxidant factors in Mouse lung fibroblasts (MLF) incubated for 24 h with TGF-β1 and various concentrations of pirfenidone (PFD) for 48 h. MLF cells were stimulated with TGF-β1 (5 ng/mL) for 24 h before PFD (100 μg/mL, 200 μg/mL and 500 μg/mL) treatment for 48 h. The mRNA expression of nuclear factor-erythroid-related factor 2 (Nrf2), [BTB (broad-complex, tramtrack and bric-a-brac) and CNC (cap‘n’collar protein) homology 1, Bach1], heme oxygenase-1 (HO-1), and glutathione peroxidase 1 (GPx1) in MLF cells was analyzed by reverse transcription-polymerase chain reaction (RT-PCR) (**a**, **b**, **c**, **d**). The protein levels of Nrf2, Bach1, Ho-1, and GPx1 in MLF cells were detected by Western blot (**e**). Data are expressed as the mean ± SD. *P < 0.05, **P < 0.01, vs. TGF-β1 group

To confirm the antioxidant capacity of PFD in MLF, we performed RT-PCR and Western blot to estimate the mRNA and protein levels, respectively, of antioxidant factors such as Nrf2, Ho-1, Gpx1, and Bach1 as a result of TGF-β1 stimulation with or without PFD in MLF (Fig. 1). After administration of 5 ng/mL TGF-β1 for 72 h, the mRNA expression levels of Nrf2, Ho-1 and Gpx1 in MLF were significantly down-regulated (Fig. 1a, c, d), while that of Bach1 was significantly increased (Fig. 1b). MLF was then treated with 100 μg/mL, 200 μg/mL, and 500 μg/mL PFD, respectively, for 48 h. RT-PCR showed that PFD rescued the inhibition effect of TGF-β1 on antioxidant gene expression and thus promoted mRNA expressions of Nrf2, Ho-1, and Gpx1 (Fig. 1a, c, d). On the other hand, PFD suppressed the up-regulation effects of TGF-β1 on Bach1 and significantly inhibited the expression of Bach1 mRNA (Fig. 1b). Furthermore, 200 μg/mL PFD substantially improved the expression of an antioxidant gene, Ho-1, as compared to that with 100 μg/mL, but was insignificant when compared to 500 μg/mL (Fig. 1c). Nrf2 and Gpx1 mRNA levels had no significant difference in various concentration (Fig. 1a and d). Western blot also showed that TGF-β1 promoted protein expressions of Nrf2, Ho-1, and Gpx1 in MLF, but significantly inhibited that of Bach1. Compared to the TGF-β1 group, PFD inhibited Bach1 protein expression in MLF and improved the protein expressions of Nrf2, Ho-1, and Gpx1. The effect on Ho-1 was remarkable under 200 μg/mL PFD (Fig. 1e). The above results indicated that PFD attenuated the inhibitory effect of antioxidative ability induced by TGF-β1 in MLF and stimulated the expression of antioxidants.

### Effects of PFD on the expressions of oxidant/antioxidant in the lung tissues of mice with BLM- induced pulmonary fibrosis

**Fig. 2:**
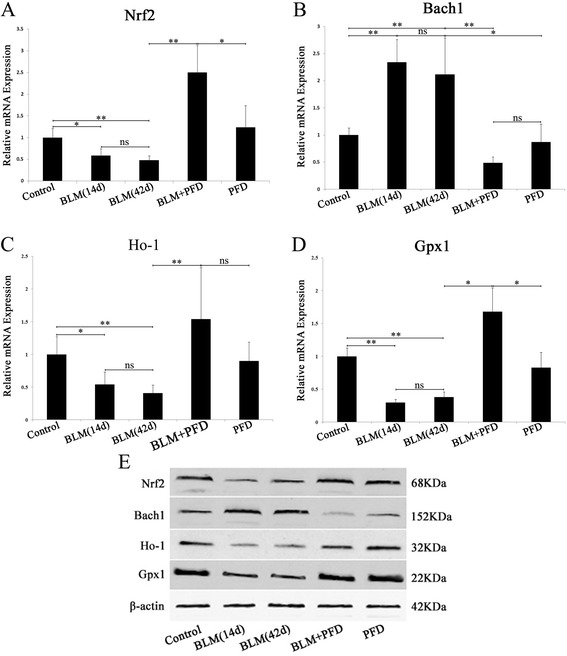
Effects of PFD on oxidant/antioxidant factors in Bleomycin (BLM)-induced pulmonary fibrosis in mice (3 in each group). BLM (5 mg/kg, intratracheally instillation for 14d) administered mice were then treated with PFD (300 mg/kg/d orally administered) for 4 weeks. Control group was intratracheally administered 50 μL 0.9% saline), only PFD group was orally administrated with 300 mg/kg PFD daily for 4 weeks. The mRNA expressions of Nrf2, Bach1, HO-1, and GPx1 in the lung were analyzed by RT-PCR (**a**, **b**, **c**, **d**); protein levels of Nrf2, Bach1, HO-1, and GPx1 in the lung were detected by Western blot (**e**). Data are expressed as the mean ± SD. *P < 0.05, **P < 0.01, vs. BLM group

To extensively explore the antioxidant mechanisms of PFD, we investigated the expression of oxidants/antioxidants in lung tissues of mice after BLM administration. RT-PCR and Western blot demonstrated that the expression levels of Bach1 mRNA and protein were significantly increased in the BLM 14d and BLM 42d groups (Fig. 2b, e), while mRNA and protein expressions of Nrf2 and the downstream antioxidants including Ho-1 and Gpx1 were significantly decreased (Fig. 2a, c, d, e). Following oral administration of PFD (300 mg/kg daily) for 4 weeks after BLM, the mRNA and protein expression levels of Bach1 were significantly down-regulated as compared to the BLM 14d and BLM 42d groups (Fig. 2b, e), and those of Nrf2, Ho-1, and Gpx1 were significantly up-regulated (Fig. 2a, c, d, e). However, the mRNA and protein expression of Bach1, Nrf2, Ho-1 and Gpx1 in PFD only group were as similar as control group (Fig. 2). The representative results suggested that the treatment with PFD promoted the expression of antioxidant factors and antioxidative characteristics in a mouse with BLM-induced pulmonary fibrosis, while such effects have not been observed in only PFD treatment.

### Inhibitory effect of PFD on fibrosis-related factors in MLF stimulated by TGF-β1 and mice with BLM-induced pulmonary fibrosis

**Fig. 3:**
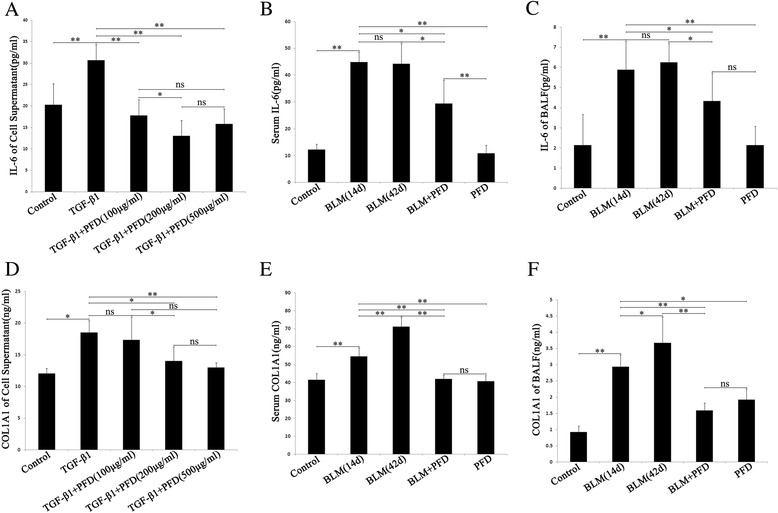
Analysis of type 1 collagen α1 (COL1A1) and interleukin-6 (IL-6) levels in the supernatant of cells, serum and bronchoalveolar lavage fluid (BALF). They were measured by enzyme-linked immunosorbent assay (ELISA). Data are expressed as the mean ± SD. **a** and **b**: COL1A1 and IL-6 levels in the supernatant of cells after TGF-β1 and various concentrations of PFD stimulation (*P < 0.05, **P < 0.01, vs. TGF-β1 group). **c** and **d**: Serum COL1A1 and IL-6 after BLM and PFD treatment (*P < 0.05, **P < 0.01, vs. BLM 14d and 42d group). **e** and **f**: COL1A1 and IL-6 levels in BALF after BLM and PFD treatment (*P < 0.05, **P < 0.01, vs. BLM 14d and 42d group)

In order to assess the therapeutic effects of PFD, fibrosis-related factors IL-6 and COL1A1 levels in MLF supernatant induced by TGF-β1, as well as those in serum and BALF of mice with BLM-induced pulmonary fibrosis were estimated. ELISA showed that TGF-β1 facilitated the expressions of COL1A1and IL-6 in MLF (Fig. 3a, b). Also, BLM up-regulated COL1A1 and IL-6 levels in serum and BALF of mouse with pulmonary fibrosis, and level of COL1A1 in BLM 42d is higher than BLM 14d group (Fig. 3c, d, e, f). Nevertheless, COL1A1and IL-6 expressions in MLF supernatant, serum, and BALF of the BLM induced mouse were significantly decreased after PFD treatment (Fig. 3), only PFD treatment had no such effects but similar as control group. This suggested that PFD inhibited the up-regulated effects of fibrosis-related factors in MLF induced by TGF-β1 and mouse with BLM-induced pulmonary fibrosis, thus exerting an anti-fibrosis effect.

### Inhibitory effect of PFD on indicators of oxidative stress in mice with BLM-induced pulmonary fibrosis

**Fig. 4:**
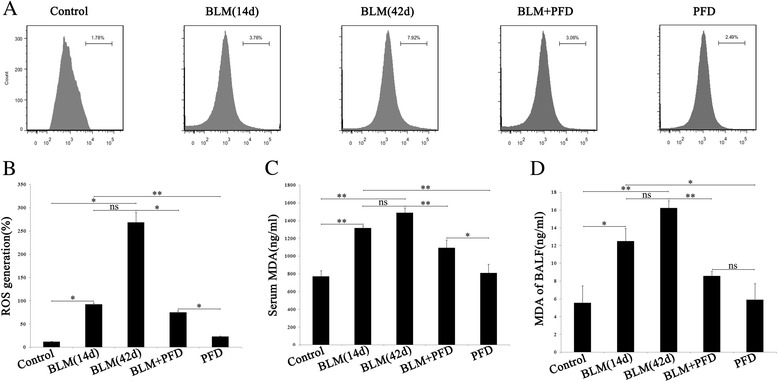
Effect of PFD on indicators of oxidative stress such as reactive oxygen species (ROS) generation and malondialdehyde (MDA) expression. **a**: ROS generation (%) in lung tissue was detected with 2,7-dichlorofluorescein diacetate (DCFDA) by flow cytometric. **b** and **c**: MDA expression in serum and BALF by ELISA. Data represent as the means ± SD. *P < 0.05, **P < 0.01, vs. BLM 14d and 42d group

To determine whether PFD exert an antioxidant effect by scavenging of free radicals in BLM-induced pulmonary fibrosis, we detected the ROS generation in the lung tissues and MDA expression in serum and BALF to estimate the degree of oxidative damage. As shown in Fig. 4a and b, ROS production in lung tissues was increased after BLM administration and more obvious in 42d than 14d, while PFD reduced BLM-induced ROS production. In contrast, there was no significant difference between the PFD only group and control group. Consistently, MDA levels of serum and BALF significantly increased in BLM 14d and 42d group compared to control group and PFD only group, while PFD suppressed BLM-induced MDA expression (Fig. 4c and d), implying a protective effect of PFD against oxidative stress.

### Histological evaluations of lung tissue after PFD treatment

**Fig. 5:**
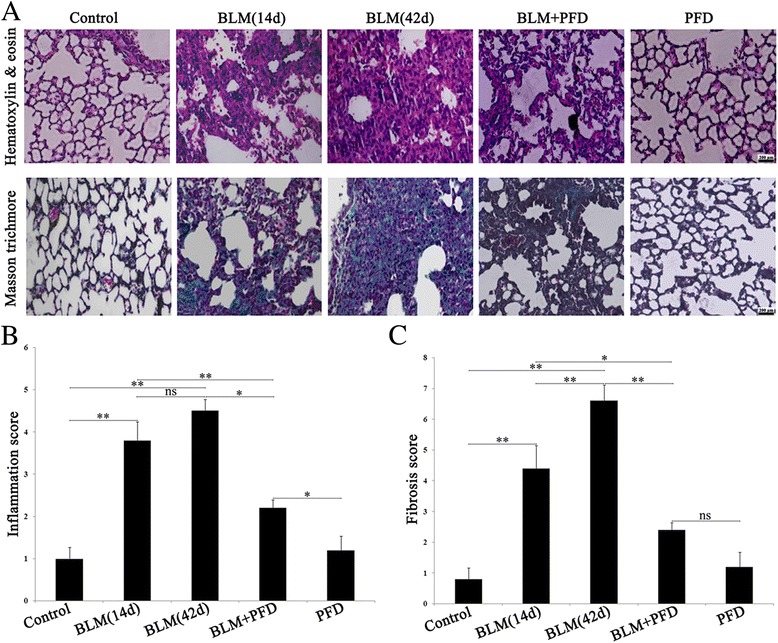
Histopathological changes of lung tissue in mice at the end point of the experiment (HE staining, ×200). BLM (5 mg/kg) intratracheally administered once for 2 weeks and then treated with pirfenidone (300 mg/kg/d orally administered) for 4 weeks. Lung histological data obtained on day 14 after BLM treatment and day 42 after PFD treatment (BLM 42 d). **a**: Lung inflammation, fibrosis, and integrity of the structure was evaluated by H&E (×200). Degree of fibrosis in lung tissues by Masson’s staining (×200). **b** and **c**: Degree of lung fibrosis was evaluated by inflammation and fibrosis scored. The data are expressed as mean ± SD,*P < 0.05, **P < 0.01, vs. BLM 14d and 42 d group

In addition to the expression of fibrosis-related factors, we further evaluated the histopathological lung alterations in mice for exploring the therapeutic effect of PFD. Inflammatory infiltration and integrity of organizational structures were observed by HE staining; the fibrosis degree of lung tissue was performed by Masson staining. The control group and only PFD group demonstrated some histological findings such as a thin alveolar wall, intact alveolar structure, normal alveolar septum, and less inflammatory cells infiltration in the pulmonary mesenchyme. After 14 days of BLM administration, alveolar edema, a significant increase in septum width and increased inflammatory cells infiltration were observed. After 42 days, the alveolus collapsed or disappeared, the structure was markedly damaged, and a large number of inflammatory cells and fibroblasts were infiltrated. Administration of PFD (300 mg/kg/day) for 4 weeks ameliorated the inflammatory infiltration and the damaged structure in lung tissue as compared to that of the BLM group. Likewise, Masson staining extensive stained blue in the lung tissue and septum after 14 and 42 days post BLM administration, suggesting a severe degree of pulmonary fibrosis in BLM group than the normal group. After PFD treatment, the blue area was decreased, and the fibrosis degree was alleviated (Fig. 5a). Similarly, at 42 days after BLM modeling, the scores of alveolitis and fibrosis were significantly increased and were substantially decreased after PFD therapy (Fig. 5b and c). The above results suggested that PFD alleviated the degree of inflammation and fibrosis in the lungs of mice with pulmonary fibrosis.

## Discussion

PFD is known to inhibit proliferation, differentiation, and the collagen synthesis of fibroblasts. It also inhibits the generation and activity of fibrosis-related cytokines TGF-β1, fibronectin, and connective tissue growth factor, thus exerting anti-inflammatory and anti-fibrosis effects that have been regarded to treat IPF [8, 23–25]. A recent in vivo study indicated that PFD suppressed chemotaxis and accumulation of fibroblasts in the lungs of a mouse with bleomycin-induced pulmonary fibrosis [9]. Importantly, the previous study reported that PFD alleviated the oxidization through its ability to suppress NADPH-dependent lipid peroxidation in liver microsome. This suggested that the anti-fibrosis activity of PFD is involved in oxidative stress [26]. Other studies also found that PFD exerted an anti-fibrosis effect by alleviating lipid peroxidation and clearing the active intracellular oxygen [11], thus demonstrating that anti-fibrosis of PFD could be associated with the oxidative stress.

Nrf2 is a central regulator for oxidation reaction, enhancing the oxidative stress in tissues and cells by stimulating the generation of downstream antioxidant substances. It indicated that stimulating Nrf2 alleviated the lung damage and suspended the progression of ILD [16, 19, 27]. Moreover, Bach1 is a competitive inhibitor for Nrf2, which also plays an essential role in the regulation of oxidative stress [15]. The defense mechanism against oxidative stress-induced damage is mediated by the transcription and protein expression of ARE and Nrf2 dependent antioxidant factors, including Ho-1, Gpx1, and others [28]. Previous evidence has shown that slicing Nrf2 down-regulated the Ho-1 expression and further promoted the transformation from fibroblasts to myofibroblasts [29]. Furthermore, it was also found that the activity and expression of Gpx1 and Ho-1 were significantly decreased in damaged lung of a mouse with bleomycin-induced pulmonary fibrosis [18, 30], suggesting that the decline of Nrf2 dependent antioxidants promoted the formation of fibrosis. Intriguingly, Bach1 competitively inhibits the interaction between Nrf2 and ARE, leading to the suppression of ARE-dependent gene [15]. However, the influence of Bach1 on the transcription and expression of ARE-dependent genes is yet controversial [31–33]. Additionally, whether PFD exerts antioxidation and anti-fibrosis effects by regulating Nrf2/Bach1 equilibrium and expression of Nrf2-dependent antioxidants, remain unclear.

The amount of extracellular matrix generated by fibroblasts and the transformation into myofibroblasts is one of the principal characteristics of IPF [34]. TGF-β1 is one of the key factors for generation of pulmonary fibrosis that promote proliferation and transformation of lung fibroblasts and deposition of extracellular matrix [4]. BLM improves the production of oxygen radical, damage of alveolar epithelium, and proliferation of fibroblasts, further leading to pulmonary fibrosis [35, 36]. In the present study, we found that the mRNA and protein expressions of Nrf2 and down-stream antioxidants such as Ho-1 and Gpx1 were significantly decreased in the MLF induced by TGF-β1 and lung tissue of mouse induced by BLM. However, the mRNA and protein expressions of Bach1 were significantly increased, demonstrating that the equilibrium state of Nrf2/Bach1 and Nrf2-dependent antioxidant factor were involved in the pathogenesis of IPF. Our previous study supported that and Bach1 knockout by siRNA enhanced the expression of Nrf2 dependent antioxidants in the TGF-β1 induced MLF and in mice of BLM induced pulmonary fibrosis [20]. Take together, the overexpression of Bach1 may be associated with the pathogenesis of IPF by affecting the antioxidant/oxidant balance. Nevertheless, following varied PFD administration, the mRNA and protein expression levels of Nrf2, Ho-1, and Gpx1 in MLF were significantly increased, but in Bach1 were significantly decreased, as compared with the TGF-β1 stimulated group. We also found that the promoting effect on Ho-1 was significant with 200 μg/mL PFD. In addition, present study showed that the mRNA and protein expression levels of Nrf2, Ho-1, and Gpx1 in mouse pulmonary fibrosis induced by BLM were significantly increased, but in Bach1 were significantly decreased with PFD treatment for 4 weeks. Our results further suggested that PFD regulated the equilibrium of Nrf2/Bach1 and expression of a Nrf2-dependent antioxidant factor, further mitigating the inhibitory effect of antioxidant level in TGF-β1 induced MLF and in mice with BLM-induced pulmonary fibrosis, thus facilitating the antioxidant effect.

Several fibrosis-related factors are involved in the pathogenesis and progression of IPF, including TGF-β1, tumor necrosis factor-α (TNF-α), IL-6, and COL1A1 by promoting the proliferation of fibroblasts and transformation to myofibroblasts, leading to huge deposition of extracellular matrix [2]. Here, our results demonstrated that the expressions of fibrosis-related cytokines IL-6 and myofibroblast markers COL1A1 were significantly increased in the supernatant of TGF-β1 induced MLF, serum, and BALF in mice with BLM-induced pulmonary fibrosis, especially in BLM 42d group. However, after PFD treatment, the levels of COL1A1and IL-6 were significantly decreased, indicating that PFD inhibited the production of a pro-fibrosis factor and the development of lung fibrosis. In addition, to determine whether PFD inhibits BLM-induced pulmonary fibrosis by scavenging free radicals, we measured the ROS generation in the lung tissues and MDA level in serum and BALF to evaluate the changes in BLM-induced oxidative damage. Excessive ROS induced by oxidative stress contributes to pulmonary fibrosis by accelerating epithelial-mesenchymal transition (EMT), infiltrating of inflammatory cell and collagen accumulation [37]. MDA, a reactive carbon compound, is regarded as one of key indicator of oxidative stress [38]. Consistent with previous studies [39, 40], our results showed that ROS generation and MDA expression there was a significant increase in BLM administered mice and more obvious in 42d than 14d. Moreover, PFD treatment showing the significant decrease in ROS and MDA levels in BLM-induced mice imply that PFD is beneficial in regulating the equilibrium of oxidant-antioxidant.

In order to further explore the antioxidant and anti-fibrosis effect of PFD, we elucidated the lung histopathological changes in mice of lung fibrosis induced by BLM with or without PFD. Following 2 weeks of BLM administration in present experiment, the alveolus collapsed or disappeared, the septum width was significantly increased, and extensive inflammatory infiltration, that is in accordance with the pathological alterations during pulmonary fibrosis. Similarly, the results of Masson showed that there was a larger blue staining area (fibrillation) and destruction of the alveolar structure after BLM administration, complying to the above [41]. The mouse modeling with IPF was thus successfully established and thus was chosen as the intervention time of PFD. After BLM d42, inflammatory infiltration was similar as d14 while the degree of fibrosis and structural damage had no obvious difference because of self-resolving of BLM- induced lung fibrosis and inflammation repair prior to fibrosis reversal. Previous study suggested that bleomycin model was a self-resolving model of lung fibrosis and fibrosis was resolved over time after a peak between d14 and d28 [42]. But in the present study, we found that a self-resolving of bleomycin- lung fibrosis from d28 to d42 expressed as alleviated inflammation, and reversible repaired of fibrosis and structural damage may take longer. It is possible that our model of pulmonary fibrosis differs from previous studies in the repair time of fibrosis because of the well-known heterogeneity of fibrosis development in that mouse model.

However, after PFD therapy for 4 weeks, the inflammatory cells infiltration and the damaged structure of lung tissue were alleviated as compared to the BLM group (d42), suggesting that PFD relieves the pathological progression of IPF.

Based on the antioxidant and anti-fibrosis effects, PFD was used to treat MLF induced by TGF-β and mouse with pulmonary fibrosis. This demonstrated the influence of PFD on Nrf2/Bach1 equilibrium and expression of Nrf2-dependent antioxidants in TGF-β induced MLF and mice with BLM-induced pulmonary fibrosis. The present evidence showed that PFD promoted the expression of antioxidant factors by reversing the inhibitory effect of antioxidant ability induced by TGF-β1 and BLM in vitro and in vivo. Moreover, such antioxidant effect was realized by regulating the equilibrium of Nrf2/Bach1 and its down-stream antioxidants. Also, PFD inhibits the up-regulation of fibrosis-related factors such as COL1A1and IL-6 induced by TGF-β1 and BLM along with indicators of oxidative stress such as ROS and MDA level induced by BLM, alleviates the inflammatory infiltration and pathological damage of pulmonary fibrosis, while only PFD treatment for wild-type mice do not display these effects. It provides the experimental evidence for the underlying antioxidant mechanism of pirfenidone treatment as well as the novel approach for pathogenesis and therapeutic targets of IPF.

## Conclusions

In summary, we draw the conclusion that the antioxidant pathogenisis of prifenidone in pulmonary fibrosis in BLM-induced mice via the regulation of Nrf2/Bach1 balance that resulted in inhibition of Bach1 and promotion of Nrf2 or Nrf2 dependent antioxidants. In light of the essential role of oxidative stress on IPF, our study explore the underlying mechanism of Nrf2/Bach1 balance responsible for pathogenesis of IPF and antioxidant therapy of prifenidone in vivo and in vitro. Such study has the potential to affluent the evidence of prifenidone therapy and propose the possibility of antioxidant therapy for IPF.

## Additional files

Additional file 1: Table S1. Primer information of RT-PCR. (PDF 9 kb)

Additional file 2: Table S2. Original data of RT-PCR in animal experiment. (XLS 43 kb)

Additional file 3: Table S3. Original data of RT-PCR in MLF. (XLS 28 kb)

Additional file 4: Table S4. Original data of ELISA in cell supernatant, serum and BALF (XLS 61 kb)

Additional file 5: Table S5. Original data of ROS expression. (XLS 25 kb)

## Abbreviations

ANOVA: One-way analysis of variance
ARE: Antioxidant response element
BACH1: BTB (broad-complex, tramtrack and bric-a-brac) and CNC (cap‘n’collar protein) homology 1
BALF: Bronchoalveolar lavage fluid
BLM: Bleomycin
N﻿r﻿f2 COL1A1: Nuc﻿lear factor-erythroid-related factor ﻿2 Type 1 collagen α1
DCFH-DA: 2′,7′-dichlorofluorescin diacetate
DEPC: Diethylpyrocarbonate
ELISA: Enzyme linked immunosorbent assay
GPx1: Glutathione peroxidase 1
Ho-1: Heme oxygenase-1
IL-6: Interleukin-6
IPF: Idiopathic pulmonary fibrosis
MDA: Malondialdehyde
MLF: Mouse lung fibroblasts
PFD: Pirfenidone
ROS: Reactive oxygen species
RT–PCR: Reverse transcription-polymerase chain reaction
TGF-β1: Transforming growth factor-β1
