# Supplementary material for: Pirfenidone attenuates bleomycin-induced pulmonary fibrosis in mice by regulating Nrf2/Bach1 equilibrium
Source: BMC Pulm Med. 2017 Apr 18;17:63. doi: 10.1186/s12890-017-0405-7 (PMC5395978; doi:10.1186/s12890-017-0405-7)
Supplement: Supplementary file 1 — Primer information of RT-PCR. (PDF 9 kb) [file 12890_2017_405_MOESM1_ESM.pdf]

Table S1. PCR primers sequence

Nrf2	Forward (5'- 3')	GTGGTTTAGGGCAGAAGG
	Reverse (5'- 3')	TCTTTCTTACTCTGCCTCTA
Bach1	Forward (5'- 3')	ACAGGGCTACTCGCAAA
	Reverse (5'- 3')	GTCATCTCCCAGGCTAATC
HO-1	Forward (5'- 3')	GACAGAAGAGGCTAAGACCGC
	Reverse (5'- 3')	TGACGAAGTGACGCCATCT
GPx1	Forward (5'- 3')	GCACATCTACCACGCAGTCA
	Reverse (5'- 3')	AGAGTCTCAAGAACATCGCCT
GAPDH	Forward (5'- 3')	AAGACCCAGAAATGAAC
	Reverse (5'- 3')	TCTACACGATAACAACCA
